# The Karachi intracranial stenosis study (KISS) Protocol: An urban multicenter case-control investigation reporting the clinical, radiologic and biochemical associations of intracranial stenosis in Pakistan

**DOI:** 10.1186/1471-2377-9-31

**Published:** 2009-07-15

**Authors:** Ayeesha Kamran Kamal, Fawad Taj, Babar Junaidi, Asif Rasheed, Moazzam Zaidi, Muhammed Murtaza, Naved Iqbal, Fahad Hashmat, Syed Vaqas Alam, Uzma Saleem, Shahan Waheed, Lajpat Bansari, Nabi Shah, Maria Samuel, Madiha Yameen, Sobia Naz, Farrukh Shahab Khan, Naveeduddin Ahmed, Khalid Mahmood, Niaz Sheikh, Karim Ullah Makki, Muhammad Masroor Ahmed, Abdul Rauf Memon, Mohammad Wasay, Nadir Ali Syed, Bhojo Khealani, Philippe M Frossard, Danish Saleheen

**Affiliations:** 1 Stroke Service, Division of Neurology, Department of Medicine, Aga Khan University Hospital, Stadium Road, Karachi-74800, Pakistan; 2 Stroke Center, Department of Neurology, Liaquat National Hospital, Institute for Post Graduate Medical Studies and Health Sciences, Stadium Road, Karachi-74800, Pakistan; 3 Department Of Medicine, Dow University of Health Sciences, Civil Hospital Karachi, Baba-e-Urdu Road, Karachi, Pakistan; 4 Department of Public Health and Primary Care, University of Cambridge, UK; 5 Center for Non-Communicable Diseases, Karachi, Pakistan

## Abstract

**Background:**

Intracranial stenosis is the most common cause of stroke among Asians. It has a poor prognosis with a high rate of recurrence. No effective medical or surgical treatment modality has been developed for the treatment of stroke due to intracranial stenosis. We aim to identify risk factors and biomarkers for intracranial stenosis and to develop techniques such as use of transcranial doppler to help diagnose intracranial stenosis in a cost-effective manner.

**Methods/Design:**

The Karachi Intracranial Stenosis Study (KISS) is a prospective, observational, case-control study to describe the clinical features and determine the risk factors of patients with stroke due to intracranial stenosis and compare them to those with stroke due to other etiologies as well as to unaffected individuals. We plan to recruit 200 patients with stroke due to intracranial stenosis and two control groups each of 150 matched individuals. The first set of controls will include patients with ischemic stroke that is due to other atherosclerotic mechanisms specifically lacunar and cardioembolic strokes. The second group will consist of stroke free individuals. Standardized interviews will be conducted to determine demographic, medical, social, and behavioral variables along with baseline medications. Mandatory procedures for inclusion in the study are clinical confirmation of stroke by a healthcare professional within 72 hours of onset, 12 lead electrocardiogram, and neuroimaging. In addition, lipid profile, serum glucose, creatinine and HbA_1C _will be measured in all participants. Ancillary tests will include carotid ultrasound, transcranial doppler and magnetic resonance or computed tomography angiogram to rule out concurrent carotid disease. Echocardiogram and other additional investigations will be performed at these centers at the discretion of the regional physicians.

**Discussion:**

The results of this study will help inform locally relevant clinical guidelines and effective public health and individual interventions.

## Background

Stroke is the third leading cause of death worldwide and about two-thirds of all strokes occur in developing countries. [1] Intracranial stenosis (ICS) due to atherosclerosis of the large arteries is the most common cause of stroke among Asians as well as African and Hispanic populations.[2-4] In contrast, Caucasians suffer from stroke most commonly due to extracranial large artery disease, particularly involving the carotid bifurcation. [3] Asians make up approximately half of the world's population and therefore ICS is probably the most common cause of stroke in the world.

### Current therapeutic strategies for ICS

The prognosis of stroke due to intracranial stenosis is poor with a recurrence rate of 38% at two years.[5] The warfarin aspirin intracranial stenosis (WASID) trial showed that despite anticoagulation with warfarin or use of high dose aspirin, the rate of re-emergent stroke is high in the territory of the affected artery.[6] The WASID trail further revealed that the use of warfarin with INR maintained at 2–3 was not superior to high dose aspirin at preventing the primary end point of stroke due to intracranial atherosclerosis.[6] Warfarin did reduce the risk of the primary end point in patients with basilar artery stenosis. However, there was no reduction in stroke in the basilar artery territory or benefit for vertebral artery stenosis or posterior circulation disease in general.[7] Cilostazol, a phosphodiesterase inhibitor that reduces re-stenosis rate after coronary angioplasty and stenting, has recently been reported in an Asian study to significantly reduce the risk of re-stenosis in patients with intracranial stroke.[8] While this study is promising it lacks sufficient follow up because these lesions are dynamic and exhibit spontaneous regression.

Surgical interventions have also been studied without favourable outcomes. The EC/IC bypass study was an extensive, multi-centre, randomized trial that showed that anastomosis of the superficial temporal artery to the middle cerebral artery for the prevention of recurrence of stroke actually leads to poorer functional outcomes in treated patients.[9,10]Presently, patients who fail to benefit from medical therapy are considered candidates for stent placement in the intracranial vessels. A multi-center, nonrandomized, prospective feasibility study evaluated the NEUROLINK system for treatment of vertebral or intracranial artery stenosis. This study showed that the re-stenosis rate among participants was 35 percent.[11] This high rate is a cause for concern but this study and other such studies using drug eluting stents do show the technical feasibility of such procedures.[12] Presently, such interventions for intracranial stenosis are considered experimental.[13] In addition, their cost limits their widespread use in resource poor settings.

### Current Biochemical Associations of Intracranial Stenosis

Stroke due to intracranial atherosclerosis is associated with atherosclerotic plaque formation in other parts of the body particularly the aorta.[14] There is 50% likelihood of coronary artery disease in patients with intracranial stenosis.[15] In addition, there is evidence that inflammation is an important determinant of atherosclerosis and stroke.[16] Its contribution seems to be higher especially in intracranial stroke. The inflammatory mechanisms at play include endothelial dysfunction, leukocyte migration, extra-cellular matrix degradation, and platelet activation.[17] Hence, there is increasing interest in inflammatory biomarkers of intracranial stenosis that may help determine its pathophysiology and provide clinicians with methods to quantify inflammation, to predict the risk of recurrent atherothrombosis and its clinical sequelae and to develop optimal therapeutic strategies.[18,19] C Reactive Protein (CRP) is a viable inflammatory biomarker which is activated by cytokines and has a pivotal role in the development and progression of atherosclerosis by induction of endothelial dysfunction [20], promotion of foam cell formation [21], inhibitions of endothelial progenitor cell survival and differentiation [22] and activation of complement in atherosclerotic plaque intima.[23] Increased CRP levels strongly predict risk for new ischemic events in primary symptomatic intracranial large-artery stroke.[24] Acute increases in the levels of Von Willibrandt factor (vWF) may be another indicator of stroke as its levels in the serum may reflect endothelial cell activation which is a necessary step in the initiation of inflammation.[25] The expression of matrix metalloproteinases (Mumps) is highly increased in atherosclerotic plaques [26] and these include MMP-1, MMP-2, and MMP-9. They are believed to play a role in the degradation of the fibrous cap of the atheroma leading to its instability.

Insulin dependent diabetes mellitus and metabolic syndrome are independently associated with a greater likelihood of intracranial large-artery atherosclerosis and diabetic patients have a significantly higher number of diseased vessels compared to disease free individuals.[27,28] Dyslipidemia, high lipoprotein A levels [29], smoking and decreased level of endostatin (an angiogenesis promoter) are all associated with intracranial stenosis.[30]

### Gaps in Knowledge

Intracranial stenosis is a common cause of disabling stroke in Asians. However, most studies used to establish its pathogenesis and risk factors have been conducted in North America or Europe where the population is unlikely to be representative of Asians. Furthermore, the prevention and control of stroke due to intracranial stenosis is particularly challenging as effective treatment options have not been established.

### Objectives of the Karachi Intracranial Stenosis Study (KISS)

The objectives of this study are to establish risk factors and to identify clinical, biochemical and radiological predictors of intracranial stenosis in South Asia. This study will also evaluate the validity of transcranial doppler as a diagnostic modality for stroke due to intracranial stenosis in this population.[8,31]

## Methods/Design

### Study Design

An observational, prospective case-control study is being conducted in order to describe the clinical presentation and hemodynamic parameters of patients who present with stroke due to intracranial vascular stenosis. Potential risk factors of intracranial stenosis will be compared with two groups of controls one of which comprises of patients with stroke due to non-ICS etiologies and the other comprises of patients without clinical evidence of stroke.

### Study Site

Stroke patients presenting to three urban tertiary care hospitals in Karachi are included in the study.

### Sample size

The targeted sample size of the study is 500 participants including 200 cases with stroke due to ICS (Group A), 150 controls with stroke due to non-ICS etiologies (Group B) and 150 stroke-free age and gender matched controls (Group C). This sample size gives the study 95% confidence and 80% power to detect any risk factors of stroke due to ICS that have a community prevalence of at least 20% and a 1.80 times higher odds in ICS patients as compared to stroke-free controls.

### Study Cases

Men and women aged 18 years or older, who present to the emergency rooms of participating hospitals and are subsequently admitted under the medicine, neurology or intensive care service with a diagnosis of stroke due atherosclerosis, are enrolled in this study by trained medical research officers. The qualifying event for enrollment in this group is sudden neurological deficit consistent with the World Health Organization criteria for stroke. According to this criteria, stroke is clinically defined by rapidly developing signs of focal (or global) disturbance of cerebral function lasting greater than twenty-four hours (unless interrupted by surgery or death), with no apparent nonvascular cause. This definition excludes patients with transient cerebral ischemia or stroke events in cases of blood disease or brain tumours.[32] Secondary strokes caused by trauma or iatrogenesis are also excluded.[33] Furthermore, the diagnosis of stroke must be supported by evidence of stroke on magnetic resonance imaging (MRI) or non-contrast computed tomography (CT) of the brain. Finally, in order to determine that the stroke was due to intracranial stenosis, magnetic resonance angiography (MRA) is used.

### Inclusion Criteria for Cases

All patients presenting to the study center are eligible for inclusion in the study if they fulfill the following requirements:

1. Development of sudden onset neurological deficit respecting a vascular territory with sustained deficit at 24 hours verified by a healthcare professional within 72 hours after onset. Onset is defined by when the patient was last seen normal.

2. Evidence of stroke on MRI or non contrast CT scan confirmation of intracranial stenosis on MRA.

3. Age greater than or equal to 18 years.

4. Modified Rankin Score less than two prior to the presenting stroke.

### Exclusion Criteria for Cases

Patients are excluded from the study if they meet any of the following criteria:

1. Hemorrhagic stroke or stroke due to non-atherosclerotic etiology, for example, Moya Moya, carotid or vertebral dissection, venous stroke, concomitant hypercoaguable state, iatrogenic stroke or stroke due to trauma.

2. Baseline mRS of three or more prior to the presenting event.

3. Life expectancy is less than six months.

4. Alcohol or drug dependence as determined by the medical research officer.

5. Inability to consent due to disability and non-availability of a valid surrogate respondent. A valid surrogate respondent is defined as a spouse, a first degree relative living in the same house or an attendant self identified as aware of the patient's medical history, habits and behaviors.

### Controls

Two groups of controls are planned. Patients presenting with ischemic stroke due to small vessel disease or cardio-embolism according to the TOAST criteria comprise the first group.[34,35] The second control group includes participants who have never suffered a clinical cerebrovascular event (stroke or transient ischemic attacks) as determined by a locally validated Questionnaire for Verifying Stroke Free Status (QVSS).[36]

### Selection Criteria for Stroke-free Controls

In order to minimize potential selection bias, controls are selected in the following order of priority.

1. The patients' spouses or other non-blood related relatives without any neurovascular event.

2. Non first degree relatives visiting the patient.

3. Patients from out-patient departments undergoing routine health evaluation.

4. Patients from out-patient departments with minor complaints for example, refractive errors, cataracts and minor ear, nose and throat complaints.

5. Patients from surgical day care departments undergoing minor elective surgery for example, hernia repair, hemorrhoids, etc.

### Matching Criteria for Stroke-free Controls

Cases and controls are matched according to gender and age (frequency matched).

### Exclusion Criteria for Stroke-free Controls

Individuals with any of the following conditions are excluded from this group:

1. Stroke or TIA phenotype or undefined prior neurologic event.

2. History of myocardial infarction or a history of coronary artery bypass and graft surgery.

3. Inability to provide consent.

4. Pregnancy.

5. Chronic conditions with major organ dysfunction such as malignancy, chronic renal failure, tuberculosis or chronic hepatitis.

### Study Procedures

Patients with suspected intra-cranial large artery ischemic stroke admitted to the participating hospitals are evaluated by physicians according to current standards for care. The evaluation includes history taking, complete physical examination, radiology including CT or MRI of the brain and MRA of the head as well as routine blood tests for evaluation of stroke patients. A standardized, structured interview is conducted and investigation results are recorded. Demographic, medical, social, and behavioral variables are determined along with baseline medications. Anthropometry is performed using standardized equipment calibrated on a daily basis. Mandatory tests include 12 lead echocardiogram (EKG), neuroimaging (CT or MRI) and blood tests including lipid profile, serum glucose and HBA_1C _measurements. Ancillary vascular imaging includes carotid ultrasound and MR or CT angiogram to rule out concurrent carotid disease. Additional testing is at the discretion of the treating physician at these centers. Controls undergo the same standard interview and biochemical investigations.

**Figure 1:**
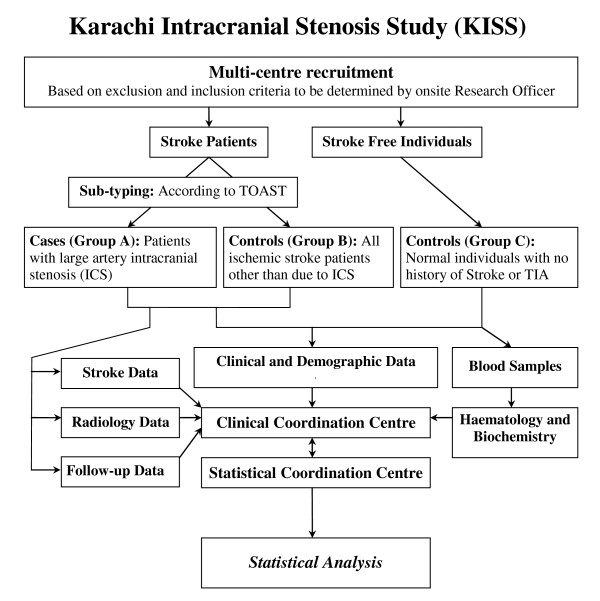
**Karachi Intracranial Stenosis Study; a summary of the study procedures**.

All clinical data, biological samples and radiological images are sent to the central study site where a cerebrovascular neurologist reviews the data and subtypes the strokes in all participants according to the TOAST criteria. A summary of the study procedures is presented in Figure 1.

### Data Collection Instrument

The following data is recorded in the data collection instrument:

1. Sociodemographic data (age, gender, and ethnicity) that is later coded for confidentiality and analysis.

2. Hospitalization information for the event and pre-hospital delays, with functional status prior to event.

3. Risk Factor Information, using internationally accepted definitions of hypertension [37], diabetes mellitus [38], smoking status, dyslipidemia [39], antecedent family history of any vascular event, presence of diabetic nephropathy, carotid stenosis, atrial fibrillation, *ghutka*(oral local tobacco) use, alcohol use and use of alternative healthcare

4. Anthropometric measurements including height, weight, waist-hip ratio, leg length, arm span and body mass index.

5. Electrocardiogram records are archived directly while all radiologic films are captured as digital images and archived on a central imaging databank.

6. Procedures carried out during hospital stay including use of antiplatelets within 24 hours, smoking cessation counseling, early mobilization, use of recombinant tissue plasminogen activator, dysphagia screening and avoidance of aspiration are specifically recorded. These variables evaluate the basic tenets of stroke care based on internationally validated data.[13,40,41]

7. In-hospital complications are noted. These include neurological complications such as recurrence of stroke, epileptic seizures and stroke progression; infections involving respiratory and urinary tracts; and complications of immobility such as falls, bedsores, deep vein thrombosis and pulmonary embolism.

### Transcranial Doppler

Transcranial Doppler ultrasound will be performed in all patients with ischemic stroke included in the study. The sensitivity and specificity of TCD will be evaluated in this population as a screening test for the presence of hemodynamically significant intracranial stenosis as identified by MRA.

### Follow Up

The enrolled cases and controls from both groups will be followed up at one, three and six months through a telephonic assessment. The objectives of the follow-up include:

1. To determine the development of delayed complications including but not limited to dementia, depression, emotionalism, frozen shoulder and post stroke pain syndrome.

2. To determine functional status using published scales (Barthel, Modified Rankin Score).

3. To determine if case fatality has taken place. For those patients who die between the periods of the follow up, a sensitive verbal autopsy shall be performed.[42]

### Data Management and Analysis

The data will be entered on Statistical Package for Social Sciences (SPSS) Version 16 and analyzed using STATA version 10. Univariate and multivariate analysis will be used to determine significance of the results obtained.

### Ethical Considerations

The study protocol has been approved by the Ethical Review Committee of all participating sites. No financial incentives are provided to any study participant. Written informed consent and verbal assent is given by all participants or their surrogate prior to the interview.

## Discussion

Intracranial stenosis may be the most common cause of stroke in the developing world. Pakistan has an exceedingly high prevalence of modifiable risk factors for stroke. Hypertension afflicts one-third of those over 45 years of age and one-fifth of those over 15 years.[43] The National Health Survey of Pakistan revealed that diabetes mellitus is present in 35% of people older than 45 years.[43] The overall prevalence of obesity is 28% in women and 22% in men while the prevalence of tobacco use is 33% in men and 4.7% in women.[44,45] This data suggests that Pakistan is on the cusp of a stroke epidemic as the population undergoes a demographic shift.[46]It is imperative to have cost effective and locally relevant solutions as a response to stroke. Clearly, optimal preventive or therapeutic strategies depend upon informed decisions. The results of this study will serve to bridge gaps in knowledge of stroke due intracranial stenosis in developing countries. In addition, this study will evaluate the use of TCD as an accessible screening tool for intracranial stenosis.

## List of abbreviations used

ICS: Intracranial stenosis; EKG: ElectrocardiogramTCD: Transcranial doppler; HBA1C: Haemoglobin A_1C_; MR: Magnetic resonance; CT: Computed tomography; DNA: Deoxyribonucleic acid; WASID trial: Warfarin aspirin intracranial stenosis trial; EC/IC: Extracranial/intracranial; CRP: C-reactive protein; vWF: Von Willibrandt factor; MMP: matrix metalloproteinase; MRA: Magnetic resonance angiography; MRI: Magnetic resonance imaging; TOAST: Trial of ORG 10172 in Acute Stroke Treatment; QVSFS: Questionnaire for Verifying Stroke-free Status; TIA: Transient ischemic attack; EEG:Electroencephalogram; BMI: Body mass index; mRS: Modified Rankin Score; SPSS: Statistical Package for social sciences

## Competing interests

The authors declare that they have no competing interests.

## Authors' contributions

AKK is the principal investigator for this project who conceived and secured funding for the project, wrote and reviewed this manuscript. PF is the co investigator involved in conceiving and running the project especially the biochemical assays. FT assisted in grant writing for this study.

BJ is the senior research associate for the current project and will perform all future Doppler applications. AR is the study coordinator for KISS. MZ was the study coordinator for KISS in the exploratory phase. FSK, NA, KM, NS, KUM, MA, ARM, MW, NAS, BK are the strong regional collaborators who have supported this work by supervision of all regional officers and access to all stroke patients. NI, FH, SVA, US, SW, LB are trained physicians specialized in data collection who performed the direct field work. NS, MS, MY, SN are the lab support personnel.

AR, MM, DS perform analysis and review of all statistical data and provide statistical overview and support. DS in addition performed biochemical testing. All authors read and approved the final manuscript.

## Pre-publication history

The pre-publication history for this paper can be accessed here:


